# The Future of Cervical Cancer Prevention: From “One-Size-Fits-All” to Personalized Screening

**DOI:** 10.3390/jpm13020161

**Published:** 2023-01-17

**Authors:** Mari Nygård, Ståle Nygård

**Affiliations:** Department of Research, Cancer Registry of Norway, 0370 Oslo, Norway

Cervical cancer screening represents an excellent model system for the development of personalized cancer-prevention strategies. It has a proven, strong effect on reducing the burden of cancer at the population level, and has provided a vast amount of clinical data at a personal level for over more than half a century. The recent progress in computational methodologies enables the use of this data to create algorithms and even complement clinical data with information about individual behavioral tendencies that augment the risk of cervical cancer as well as the HPV vaccination status. The promise is to design tailored actions safely, efficiently and quickly to identify individuals with high risk and to personalize cancer screening.

Mass screening with repeated cytology accompanied by the effective treatment of cervical pre-cancers has been a highly effective policy in reducing the incidence and mortality caused by cervical cancer [1]. This “one-size-fits-all” strategy was designed during the 1960s to deliver a single-screening technology, the cytology test, and a single diagnostic technology, the colposcopy, to a large segment of the female population. Cytology tests require a collection of exfoliated cells from the cervix during a pelvic examination and pathology services to diagnose abnormal cells. Pre-cancerous conditions, that will ultimately be treated, are then confirmed by histopathological examination of cervical tissue. Hence, in addition to delivering screening exams, cancer-screening programs are also dependent on effective pathology and gynecology services. These resources are seldom available in low-income countries. Globally, cervical cancer is the fourth most common cancer in females and is affecting low-income countries disproportionally [2]. Efforts to develop more effective screening programs are, therefore, highly relevant to reduce this unequally distributed cancer burden and reducing the overall cost of the screening.

Modern technologies offer far more options for cervical cancer prevention than what was possible when screening was initiated. After Harald zur Hausen (2008 Nobel Laureate in Medicine) and colleagues discovered HPV in cervical cancer tissue in the 1980s and suggested the causal link between HPV and cervical cancer, the understanding of the major cause of cervical cancer advanced rapidly [3]. Importantly, the discovery of the role of persistent carcinogenic HPV infection in cervical cancer development has led to at least two disruptive innovations: (i) the development of molecular HPV tests which replace cytology tests in screening and (ii) the development of HPV vaccines.

Since 2006, it has repeatedly been demonstrated that HPV tests are more sensitive for detecting cervical pre-cancers and cancers and that they are easier to implement on a large scale, compared to cytology tests [4,5,6,7]. HPV-negatives are unlikely to develop cancer within ten years, implying that rescreening can be extended, with the result of longer screening intervals and fewer tests overall [8]. Despite the evidence and the availability of commercial HPV assays for primary cervical cancer screenings [9], the roll-out of HPV screenings has been slow. As of July 2019, the Netherlands and Turkey were the only European countries where HPV screening was rolled out nationally [10]. The delayed uptake of new technology implies shortcomings and inefficiencies in the existing screening, resulting in missed opportunities and wasted resources. To move existing cancer screening systems towards flexibility, scalability and sustainability, more studies are needed to understand the barriers to implementing advances from research to practice.

Prophylactic vaccines are the most recent advancement in cervical cancer prevention. The vaccination against HPV16 and 18, which cause approximately 70% of cervical cancers [11], and the vaccination against HPV16, 18, 31, 33, 45, 52, and 58, which cause approximately 90% of cervical cancers [12,13], will eliminate the HPV types with the highest oncogenic potential and will change the accuracy of the screening test to detect pre-cancers [14]. In order for screening to remain a high-value public health intervention, mathematical models suggest that only one or two lifetime screens are required in a vaccinated population, instead of the current 12–15 [15,16]. To improve screening accuracy, novel biomarkers will allow for a clearer separation between women with clinically irrelevant HPV infections and those with virus-induced cell damage, which will allow for a more individual risk assessment for every screened woman [17]. The detection of methylations in the human genome and cellular markers for disease progression can further improve the estimation of cancer risks and discriminate between what screen positives are destined to progress or regress [18]. The next generation sequencing describing HPV genotype variants and point mutations in clinical materials in unprecedented detail is currently another active research area which might result in powerful applications in clinical diagnostics [19]. Finally, the role of vaginal microbiota as a biomarker of the progression and regression of HPV and HPV-related diseases is subject to extensive research [20].

The introduction of HPV vaccines, the use of HPV testing in screening, and the use of HPV typing and other biomarkers after a positive screening test, make a personalized risk assessment pertinent [21,22,23]. The concept of personalized cancer prevention is attracting increasing interest, as the screening, diagnostics, and treatment choices are increasing due to scientific discoveries. The added complexity requires computerized assistance for the proper management of population segments with unequal risk levels and challenges the conceptual and logistical framework of delivering the existing cancer screening which is designed to deliver preventive health care in the traditional “one-size-fits-all” approach.

While European guidelines recommend screening program coverage to be as high as 85% [24], a real-world screening uptake is often sub-optimal, with approximately 50% of cancers arising from the sub-optimally screened population. Only 20% of the target population in Norway had repeated screening exams at recommended intervals, whereas 8% did not attend a screening at all, and 46% were under-screened [25]. Ethnic minorities and socioeconomically challenged groups are over-represented among screening non-attenders, suggesting social disparities and inequalities [26,27,28,29]. Also, pelvic examination (i.e., visual and physical examination of reproductive organs) necessary to collect screening sample, is often perceived as a painful, embarrassing and uncomfortable [26,30]. Recently introduced self-sampling for HPV testing is a good alternative for pelvic examination and has already been successfully introduced in screening programs. Nevertheless, there is no universal, single reason explaining an aversion to screening. Typically, interventions improving screening rates are developed within the existing cultural context and communicated using layman terms. We recently reported that the gamification of important principles in cervical cancer prevention strategies improved the processing and contextualizing of the complex medical information, and resulted in significantly higher attendance to screening compared to those who were not exposed to the game [31]. This suggests that innovative, personally tailored solutions can be developed to overcome barriers and increase screening program coverage.

Finally, there are several ethical challenges associated with designing and managing a screening program, including the balance between over- and under-treatment and the optimization of expenditure from a broad public health perspective. The communication of cancer risks can be challenging, and more research is needed on how elevated risks could be communicated in a way that minimizes unnecessary stress and anxiety.

Cervical cancer screening programs are living through challenging and demanding times. Novel technologies have the potential to improve the accuracy of cervical cancer prevention, but implementation takes time. To maintain the intended harm-benefit balance in HPV-based screening, the necessary nuanced follow-up can be obtained using different biomarkers. The start age and frequency of screening intervals are expected to be different in individuals who are not vaccinated at all, vaccinated against 2/4, or 9 HPV types.

The availability of new technologies allows us to embrace a very different future which is characterized by innovations and changes in how we conduct cancer screening. The willingness to implement changes can be pushed back by the wish to retain stability and double down on elements from the past. We believe that a winning mindset acknowledges that the success of cervical cancer prevention is dependent on an intelligent combination of elements from mass screening and novel opportunities that can meet the personalized needs of the target population.

For the Special Issue *Recent Advances in Personalized Cervical Cancer Screening*, we are welcoming original research and review articles underpinning how new technologies and/or computational methods can be used to develop more personalized cervical cancer screening. This will include research on how to identify and reach people at high risk of developing (pre-)cancers, identification of novel biomarkers and risk factors, as well as intelligent combinations, e.g., via machine learning, of novel and established factors for improved risk stratification. Such research will be of great value for developing more effective cervical cancer screening programs in which the under- and over-treatment of patients is reduced.
